# Impact of interpersonal relationships and acquired capability for suicide on suicide attempts: An observational study

**DOI:** 10.1192/j.eurpsy.2022.2161

**Published:** 2022-09-01

**Authors:** S.H. Shim, J.C. Yang, S.W. Hahn, J.S. Kim, S.H. Yoon

**Affiliations:** 1 Soonchunhyang University Cheonan Hospital, Department Of Psychiatry, Cheonan, Korea, Republic of; 2 Chonbuk National University Hospital, Department Of Psychiatry, Jeonju, Korea, Republic of; 3 Soonchunhyang Univeristy, Seoul Hospital, Department Of Psychiatry, Seoul, Korea, Republic of; 4 SOON CHUN HYANG UNIVERSITY HOSPITAL CHEONAN, Psychiatry, Cheonan, Korea, Republic of

**Keywords:** interpersonal relationships, acquired capability, Suicide

## Abstract

**Introduction:**

The Interpersonal-Psychological Theory of Suicide (IPTS) specifically predicts that acquired capability, perceived burdensomeness, and low belongingness are collectively necessary and sufficient proximal causes of serious suicidal behavior. Although the interpersonal theory of suicide is clinically reliable, most previous studies have been conducted on clinical groups including suicidal ideators with no suicide attempters or including only a few suicide attempters

**Objectives:**

This study aims to investigate interpersonal needs and acquired capability for suicide through questionnaire surveys following suicide attempts in people admitted to hospitals for medical treatment.

**Methods:**

A total of 344 participants (200 depressed patients with attempted suicide, 144 depressed patients with suicidal ideation) were enrolled for this study. Depression, anxiety, emotional regulation, interpersonal needs, and acquired capability for suicide were evaluated. A model with pathways from emotional regulation difficulties and interpersonal needs to suicide attempts was proposed. Participants were divided into two groups according to the presence of suicide attempts or suicidal ideation.

**Results:**

Acquired capability for suicide mediated the path from depression to suicide attempts. In the path model, difficulties in emotional regulation and interpersonal needs predicted depression significantly. Although depression itself was not significantly related to acquired capability for suicide, depression was significantly related to acquired capability for suicide in suicide attempter group.

**Conclusions:**

Interventions with two factors affecting suicide attempts will clarify the suicide risk and contribute to finding risk factors. It will also help reduce suicide rates through interventions in the processes leading to suicide attempts by identifying variables to predict the attempts through the path to suicide attempts.

**Disclosure:**

No significant relationships.

